# Application of metabolomics in the diagnosis of breast cancer: a systematic review

**DOI:** 10.7150/jca.37604

**Published:** 2020-02-10

**Authors:** Liqing Yang, Ying Wang, Haishan Cai, Shuang Wang, Yueping Shen, Chaofu Ke

**Affiliations:** 1 Medical College of Soochow University, Suzhou 215123, P. R. China.; 2 Department of Epidemiology and Biostatistics, School of Public Health, Medical College of Soochow University, 199 Renai Road, Suzhou 215123, P. R. China.

**Keywords:** Breast Cancer, Metabolomics, Diagnosis

## Abstract

Breast cancer (BC) remains the most frequent type of cancer in females worldwide. However, the pathogenesis of BC is still under the cloud, along with the huge challenge of early diagnosis, which is widely acknowledged as the key to a successful therapy. Metabolomics, a newborn innovative technique in recent years, has demonstrated great potential in cancer-related researches. The aim of this review is to look back on clinical and cellular metabolomic studies in the diagnosis of BC over the past decade, and provide a systematic summary of metabolic biomarkers and pathways related to BC diagnosis.

## 1. Introduction

Breast cancer (BC) has become the most common cancer in women globally, accounting for nearly 2.1 million newly diagnosed cases in 2018 based on the data from Globocan 1. Previous researches have evidenced that early diagnosis and timely treatment of BC would exert a significant effect on improving the prognosis of BC patients 2. As the most widely used tumor markers for BC, neither CA 15-3 nor CEA have satisfactory sensitivities and specificities for early diagnosis 3. Although annual digital mammography (DM) has been regarded as an effective way to reduce the mortality of BC in age-appropriate asymptomatic women, the sensitivity tends to depend on tissue density and tumor growth patterns 4. Recent studies indicated that the digital breast tomosynthesis (DBT) might have the potential to replace DM in the early detection of BC, with a preponderance of sensitivity in dense breast to some extent, but an overview of these small-scale evidences came to a conclusion that it is still insufficient to confirm a shift from DBT to DM 5. Therefore, novel effective and convenient methods for early diagnosis of BC are urgently needed.

**Figure 1:**
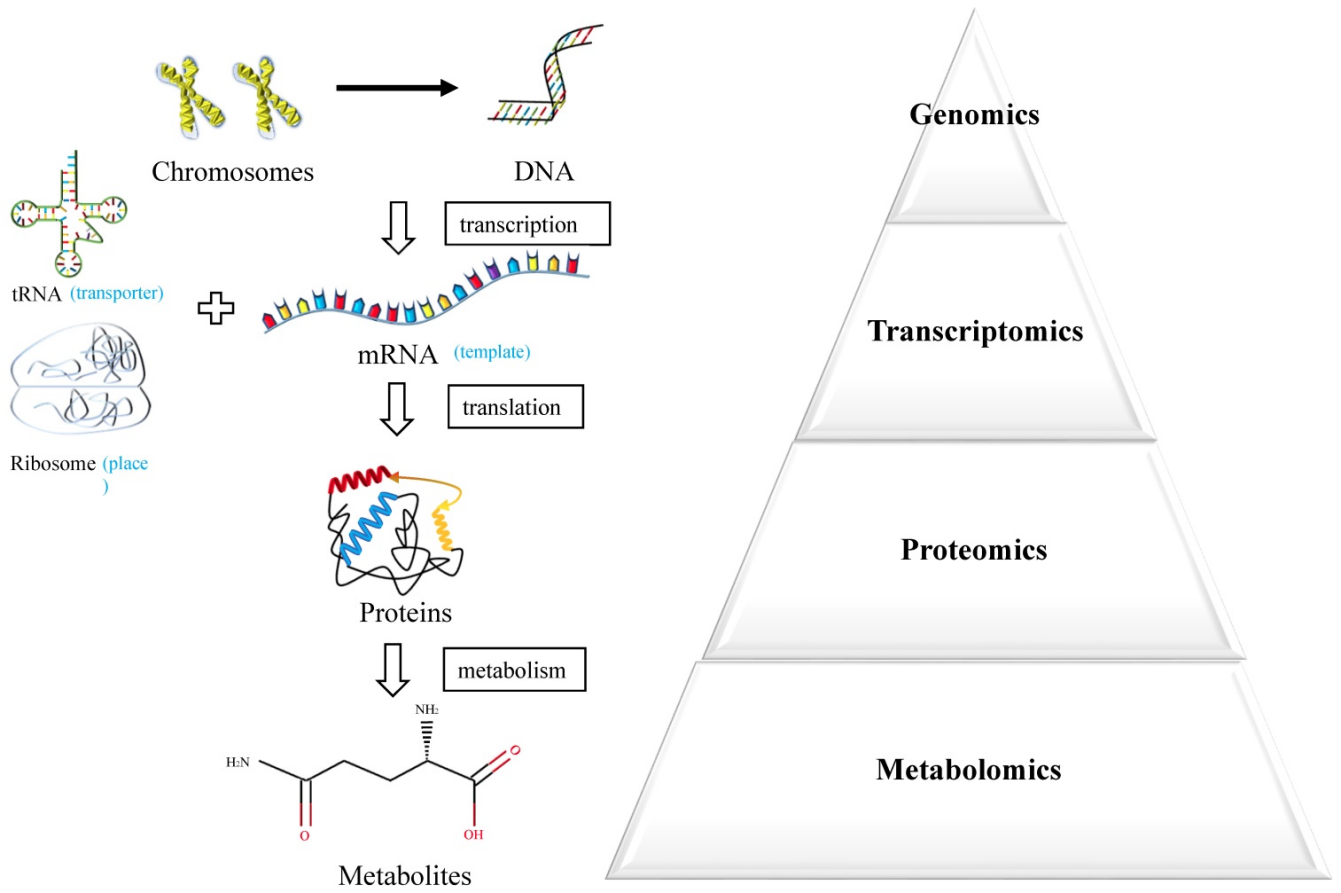
Systems biology approaches.

Metabolomics is an emerging powerful technique measuring endogenous metabolic substances in response to internal and external changes of the whole body 6 (Figure 1). The molecules produced by cancer cells during their growth could enter into the urine, blood or tissue, which underlies the potential to discover valuable biomarkers for early diagnosis 7. In recent years, the technique of metabolomics has been widely used in the discovery of biomarkers in various cancers and served as an effective approach for personalized medicine.

In this study, we performed a systematic review about the clinical and cellular metabolomic researches in the diagnosis of BC over the past decade, pursuing an overall perspective over the potential application of metabolomics in the diagnosis of BC and discovery of reliable metabolic biomarkers and pathways for BC.

## 2. Materials and Methods

### 2.1 Literature Searching

Researchers conducted an advanced retrieval on the PMC platform with the following searching strings: (“metabolomics” OR “metabolic profiling” OR “metabolic profiles” OR “metabolic biomarkers” OR “metabolome” OR “metabolic protraits”) AND (“breast” OR “mammary”) AND (“tumor” OR “tumour” OR “cancer” OR “carcinoma” OR “neoplasm”). Literatures published between 2008/01/01 and 2019/08/01 were included in the index, with a result of 12068 records. Two researchers searched the articles independently and the third one made the final decision if necessary.

### 2.2 Inclusion and Exclusion Criteria

**Figure 2:**
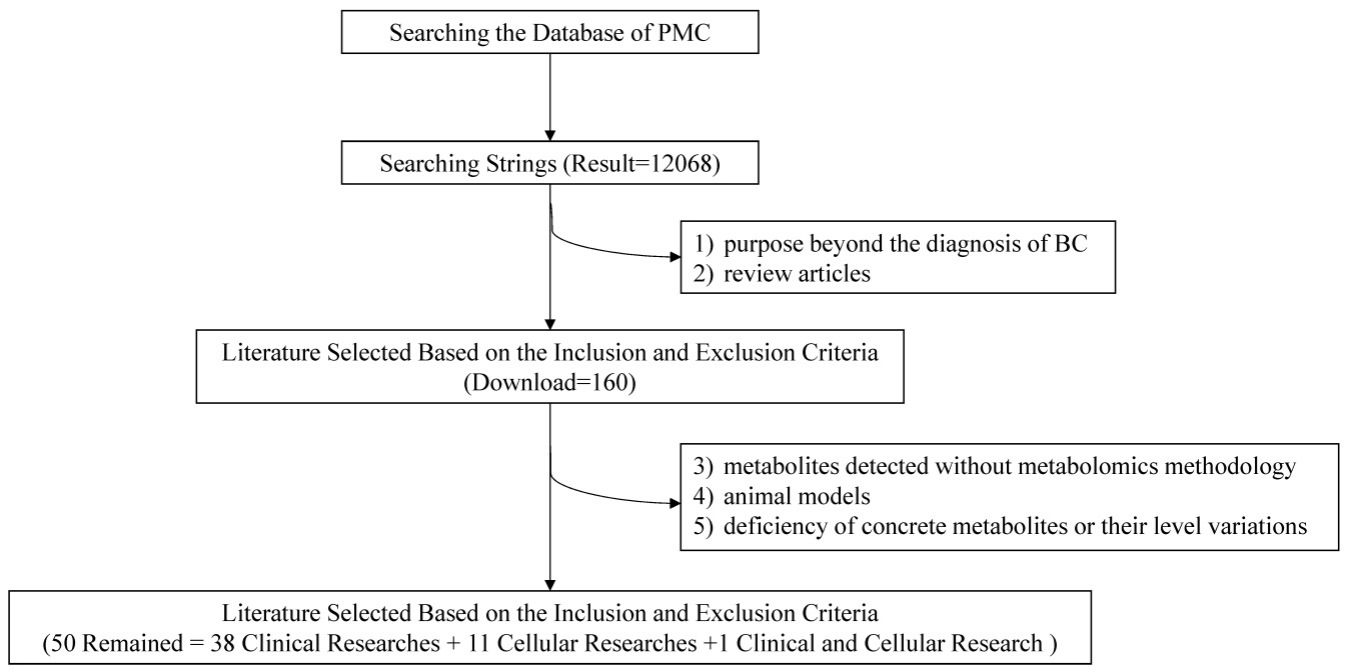
Flow chart of the literature search and selection.

To lower the missing rate of targeted records, relevant metabolomic studies were all included except the following: 1) purposes beyond the diagnosis of BC; 2) review articles; 3) metabolites detected without metabolomics methodology; 4) animal models; 5) deficiency of concrete metabolites or their level variations. Eventually, 50 items including 38 clinical researches, 11 cellular researches and 1 clinical and cellular research were remained with required information recorded (Figure 2).

### 2.3 Data Extraction

Information from selected literatures was extracted as followed.

1) Title, first author and publication year;

2) Purpose, study subjects (case and control) and analytical techniques;

3) The significant metabolites with changing trends.

### 2.4 Statistical Analysis

Frequencies on detecting instruments, biological specimens, sample sizes, study designs and repeatedly reported biomarkers were counted and graphed. The pathway analysis of metabolite markers in BC, including enrichment analysis and pathway topology analysis, was performed using the online software of *Metaboanalyst* (http://www.metaboanalyst.ca/).

## 3. Results

### 3.1 Clinical Researches

#### 3.1.1 Study Characteristics

**Figure 3:**
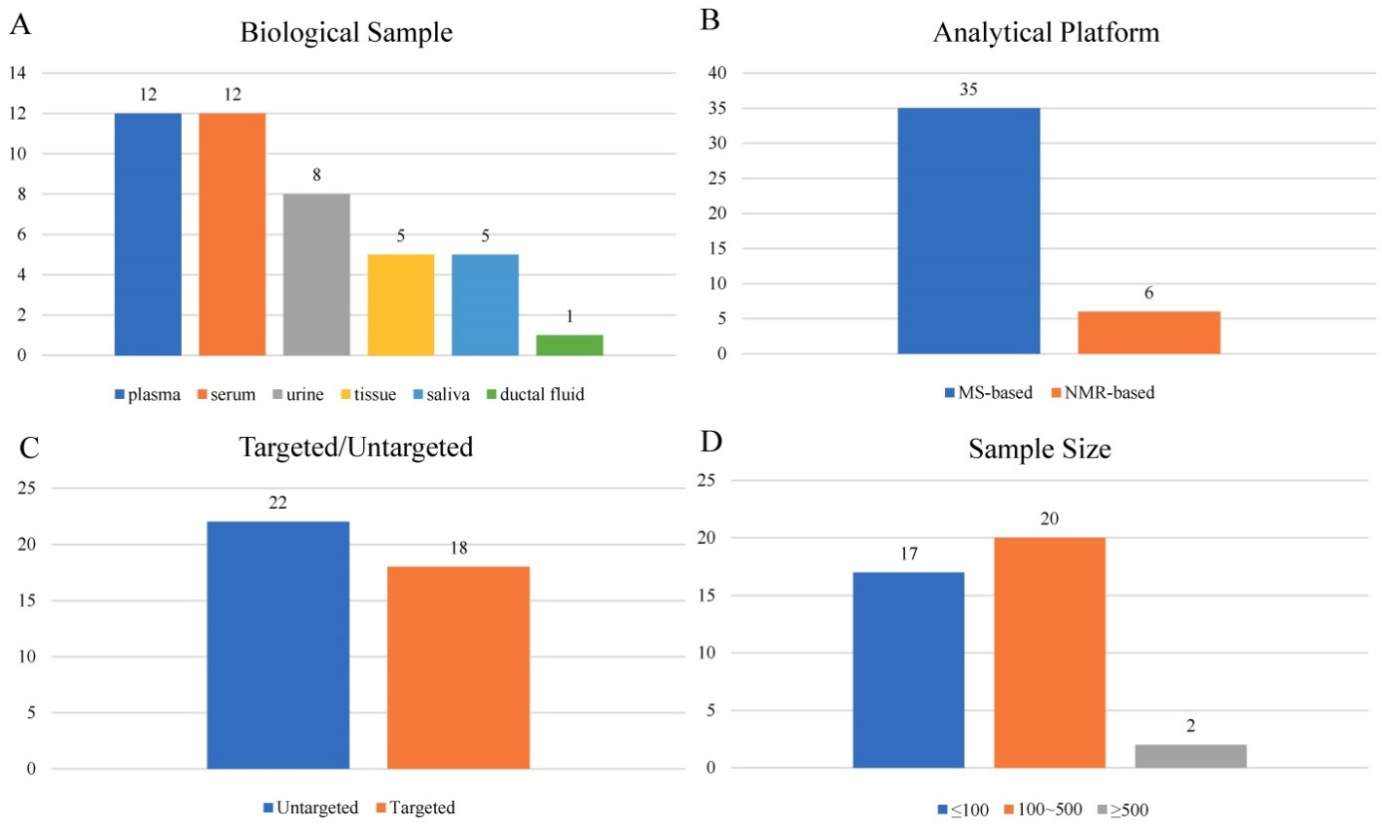
Characteristics of clinical studies.

A total of 39 articles^8^-^46^ were included in the final analysis (Supplementary Table S1), among which 22 studies were performed with blood (serum or plasma), 8 with urine, 5 with tissue, 5 with saliva and 1 with ductal fluid (Figure 3A). Mass-spectrometry based metabolomics studies reached 35 articles, while NMR was adopted in 6 studies (Figure 3B). Eighteen studies were targeted, and the other 22 studies were untargeted (Figure 3C). Study sample sizes summing the case and control varied from 3 to 1172, with only 2 studies bigger than 500 (Figure 3D).

#### 3.1.2 Analysis of High Frequency Metabolic Biomarkers

In all, 492 metabolite markers mentioned in these diagnosis-related studies were recorded. Table 1 summarized 33 metabolic biomarkers with high frequency (reported in ≥ 3 studies). Tyrosine has the highest frequency with 12 hits in total, followed by alanine reported with 11 hits. In studies focusing on the tissue 16, 18, 36, 37, 40, most significant metabolites tended to be up-regulated except glucose. Notably, changes of some fatty acids like palmitic acid, linoleic acid and stearic acid were consistently increased in the blood.

#### 3.1.3 Analysis of Metabolic Pathways

**Figure 4:**
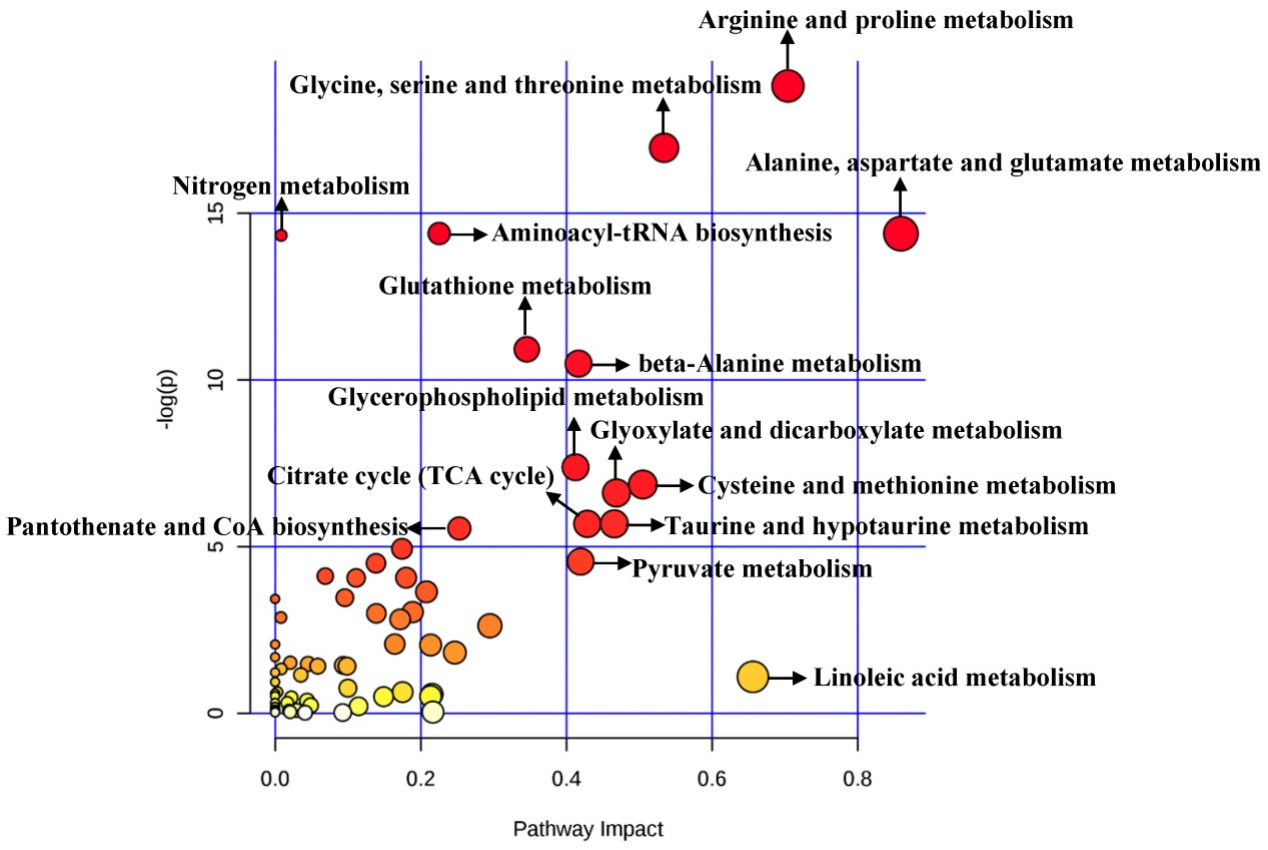
Pathway analysis for clinical significant metabolites.

A total of 492 significant metabolites were imported to *MetaboAnalyst* for the identification of involved metabolic pathways (Figure 4). Those significantly enriched pathways (raw *P*<0.005) included arginine and proline metabolism, glycine, serine and threonine metabolism, aminoacyl-tRNA biosynthesis, alanine, aspartate and glutamate metabolism, glutathione metabolism and so on (Table 2). Particularly, two pathways (alanine, aspartate and glutamate metabolism and arginine and proline metabolism**)** performed an impact of 0.85945 and 0.70435, respectively.

#### 3.1.4 Diagnostic potential of metabolite markers for discriminating BC

Twenty-two of these studies comparing BC patients with controls reported exact AUC values ranging from 0.627 to 1.000 (Table 3). In addition, Mónica Cala et al. 22 demonstrated that specific built-up biomarkers like dimethylheptanoylcarnitine and succinic acid could have a higher sensitivity and specificity (93.5%, 86.2%) than either dimethylheptanoylcarnitine (71.0%, 75.9%) or succinic acid (67.7%, 75.9%). Notably, the stage of BC patients has a certain influence on the results according to three selected studies 13, 30, 41.

### 3.2 Cellular Researches

#### 3.2.1 Study Characteristics

**Figure 5:**
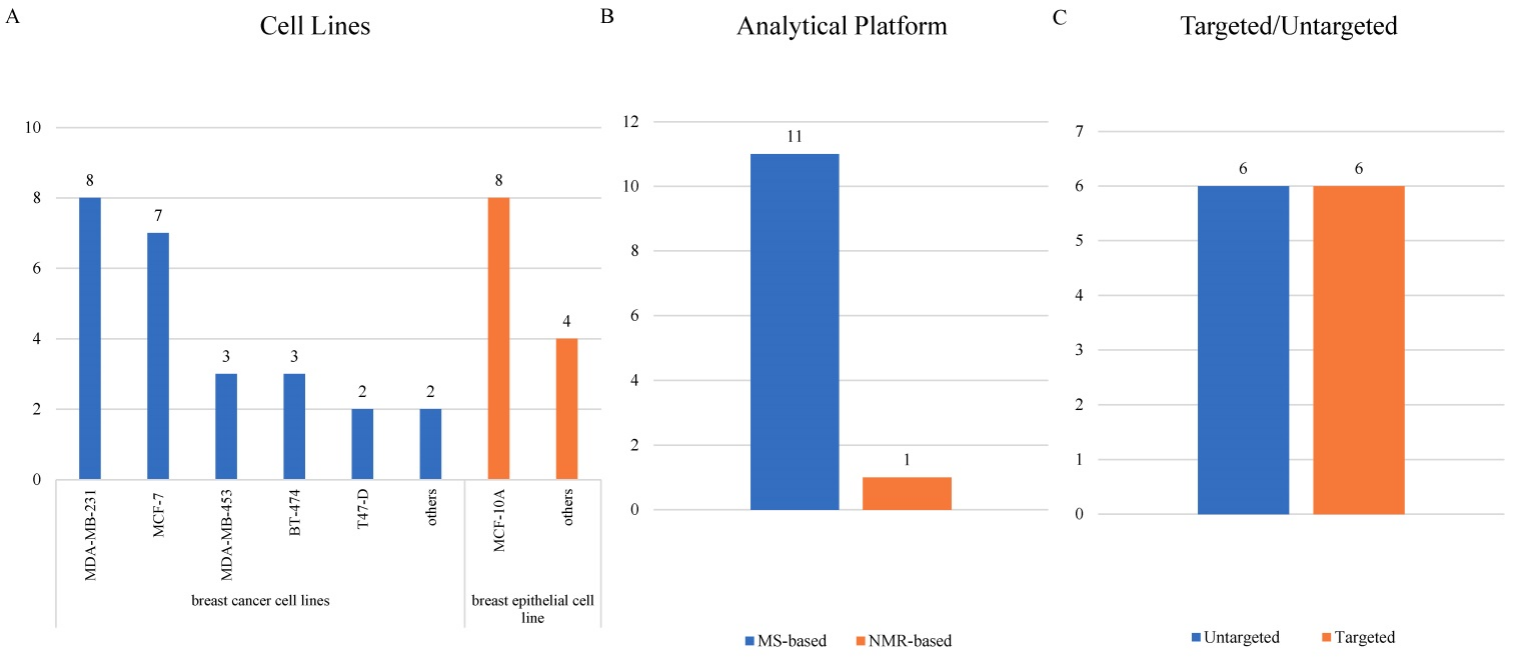
Characteristics of cellular studies.

Cellular researches were relatively fewer than clinical researches, and 12 cellular studies 32, 47-57 were ultimately included (Supplementary Table S2). MCF-10A, mentioned in 8 articles, led the most common normal cell line being studied. As for BC cell lines, MDA-MB-231 was adopted most frequently in 8 studies, followed by MCF-7 in 7 studies and both MDA-MB-453 and BT-474 in 3 studies (Figure 5A). Only one study detected metabolites using a NMR-related methodology, and the other 11 studies all adopted mass-spectrometry based metabolomics (Figure 5B). Targeted and untargeted metabolomics both accounted for 6 articles (Figure 5C).

#### 3.2.2 Analysis of High Frequency Metabolic Biomarkers

There were 84 significant metabolites in the BC cells compared with normal breast epithelial cells. Differential metabolites reported in two studies included decreased amino acids (e.g. leucine, isoleucine, valine, phenylalanine 47, 51 and glutamine 47, 57), increased lipids (e.g. phosphocholine 53, 56) and RNA metabolites (e.g. 3-methylcytidine and 5-methyluridine 49, 52). Moreover, decreased glucose was observed in two studies 53, 57 (Table 4).

#### 3.2.3 Analysis of Metabolic Pathways

**Figure 6:**
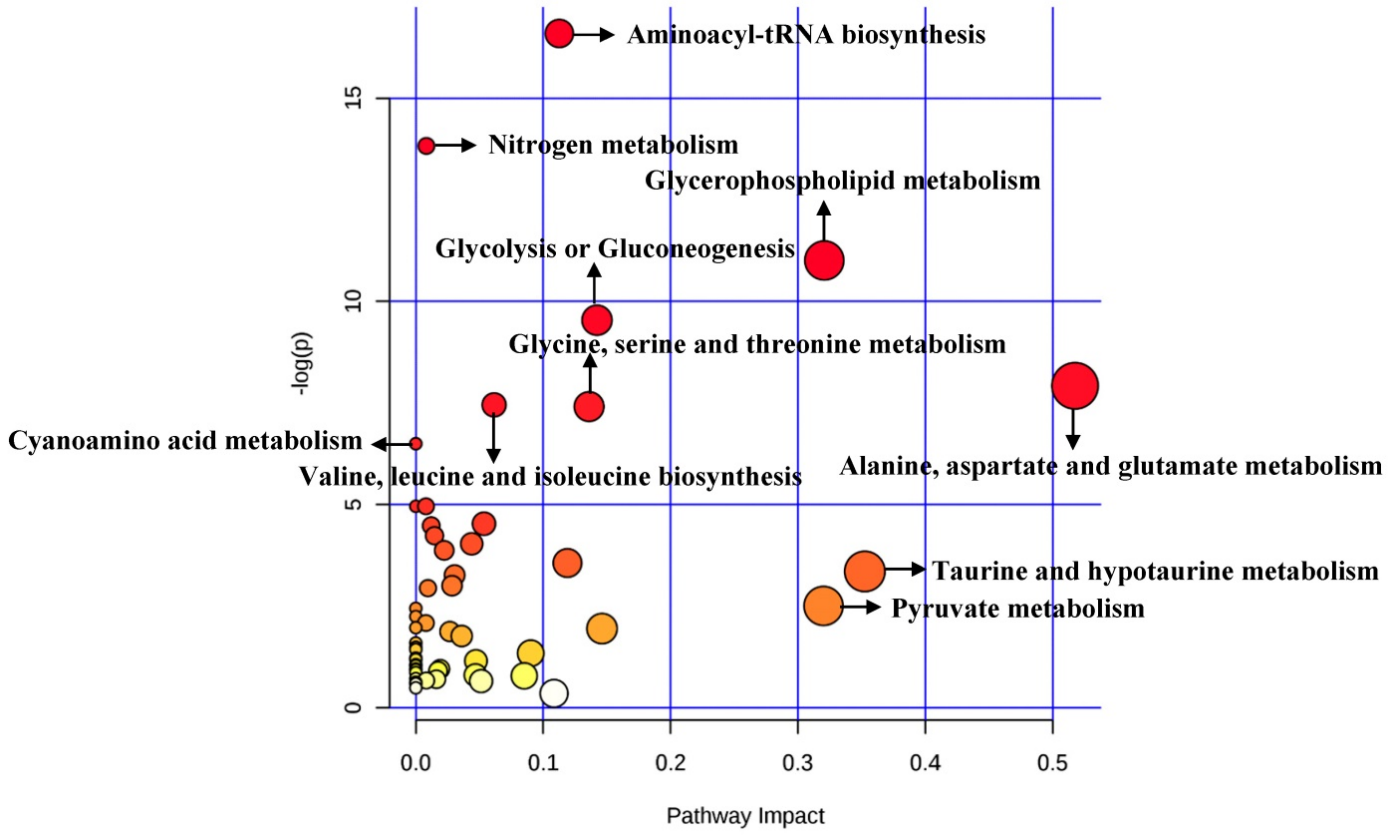
Pathway analysis for cellular significant metabolites.

A total of 84 metabolites were imported to *MetaboAnalyst* for the identification of involved metabolic pathways (Figure 6). Finally, significantly enriched metabolic pathways (raw *P*<0.005) included aminoacyl-tRNA biosynthesis, glycerophospholipid metabolism, glycolysis or gluconeogenesis, alanine, aspartate and glutamate metabolism, glycine, serine and threonine metabolism and so on (Table 5). Notably, alanine, aspartate and glutamate metabolism had the highest impact value (impact= 0.51757).

## 4. Discussion

In this study, we performed a systematic analysis of clinical and cellular metabolomic studies on BC diagnosis. As a result, a series of potential biomarkers were reported and summarized. A total of 33 high-frequency metabolites in clinical researches (reported in ≥3 studies) were listed, and some metabolic biomarkers (e.g. palmitic acid, linoleic acid, stearic acid and lipids.) showed consistent changing trends. In addition, pathway analysis revealed several important metabolic pathways for BC, particularly alanine, aspartate and glutamate metabolism with the highest impact, both in cellular and clinical studies.

When comparing clinical and cellular researches, we found that some significant metabolites (e.g. leucine, isoleucine, valine, phenylalanine, glutamine and glucose) were repeatedly reported in both types of studies. Moreover, five metabolic pathways were significantly enriched in both types of studies (FDR<0.05), including glycine, serine and threonine metabolism, aminoacyl-tRNA biosynthesis, alanine, aspartate and glutamate metabolism, nitrogen metabolism and glycerophospholipid metabolism. Along with the heterogeneity of BC, metabolites in different patients could vary based on different samples, tumor characteristics and dietary structures 58. By contrast, cellular studies have an advantage of avoiding the heterogeneity resulting from diverse samples. However, metabolites detected could also be fluctuant, which is induced by the incubation time 47, 51 and PH of mediums 48.

**Figure 7:**
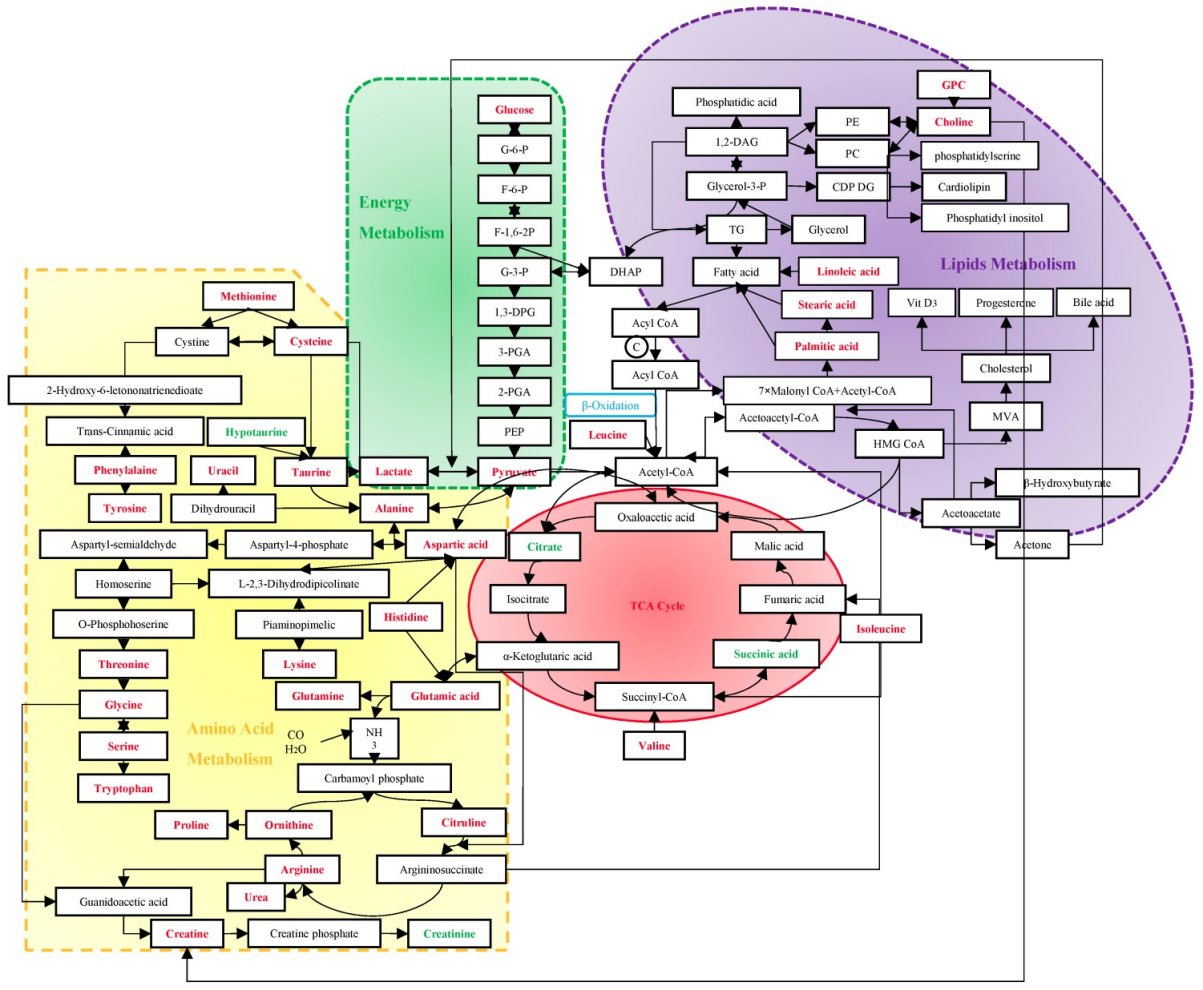
Metabolism in the diagnosis of BC. The red stand for metabolites with hits more than 3, and the green stand for 2-hit frequency metabolites. Abbreviation: G-6-P: glucose-6-phosphate; F-6-P: fructose-6-phosphate; F-1,6-2P: fructose-1, 6-bishosphate; G-3-P: glyceraldehyde-3-phophate; 1,3-DPG: 1,3-diphosphoglyceric acid; 3-PGA: 3-phosphoglycerate; 2-PGA: 2-phosphoglycerate; PEP: phosphoenolpyruvate; DHAP: dihydroxyacetone phosphate; TG: triglyceride; Glycerol-3-P: glycerol-3-phosphate; 1,2-DAG: 1,2-diacylglycerol; PE: phosphatidyl ethanolamine; PC: phosphatidylcholine; CDP DG: cytidine-5'-diphosphate 1,2-diacyl-sn-glycerol; GPC: glycerophosphocholine; MVA: mevalonic acid; HMG CoA: β-hydroxy-β-methylglutaryl- coenzyme A; TCA: tricarboxylic acid.

Screening mammography has been acknowledged as the gold standard for early detection of BC, with sensitivities of 54%~77% 59. Despite the rapid development of many imaging techniques, their utilizations are limited to the high cost and insufficient sensitivities and specificities 60. Due to the important role of immune system in the process of tumors' origin and development, tumor antigen-specific autoantibodies can be potentially applied to cancer detection as early biomarkers. However, currently used tumor markers usually have low diagnostic specificities and sensitivities as well 61. Vathany's study suggested that cancer biomarkers could be measured in serum by immunological techniques with superiorities of convenience, relative non-invasion and being tested independently by operators 61. As evidenced by the preponderant sensitivities and specificities in previous studies, metabolomics has shown advantages in the early diagnosis of BC. It is well known that an acceleration of glyconeogenesis, glycolysis and fat mobilization, and a decrease in protein synthesis, are the main metabolic changes in malignant tumors. The following discussion will expand from three basic metabolic pathways to discover their sensitivities and specificities for BC (Figure 7).

### 4.1 Energy Metabolism

Altered utilization of energy relative to normal cells caused by the proliferative tumor cells is an acknowledged hallmark of several cancers 62. In glycolysis metabolism, a decreasing trend of glucose 23, 34, 37, 53, 57 and an increasing tendency of lactate 11, 17, 21, 37, 57 were observed in BC. This phenomenon might be accounted for a shift in energy production of tumor cells with a preference to anaerobic glycolysis even in the presence of oxygen, known as the Warburg effect 63, which is a better way for the rapid acquisition of energy and self-protection by immune disruptions with an acidic environment 64. As a result of favoring the carbon source, MCF-7 cells were more likely to accomplish the complete oxidation of carbon consumption than 48R cells 57. Moreover, higher lactate levels had been found associated with lower 5-year survival rates 65. Notably, an increasing level of pyruvate can be bound up with an enhanced glycolytic activity 43, 66. A high level of anaerobic glycolysis could reduce intermediates in the TCA cycle, resulting in a suppressed TCA cycle 43, which might be one of the reasons for down-regulations of branched chain amino acids (BCAAs) in the tissue of BC including leucine, isoleucine and valine 67.

### 4.2 Amino Acid Metabolism

Former studies suggested that several decreased amino acids might be the result of excessive consumption or preferential utilization to sustain the uncontrolled growth of BC cells 18, 22, 24, 27, 42, 47, 51, 68. Based on clinical metabolomic studies, tyrosine and alanine shared the highest frequency with 12 hits, indicating they might be sensitive metabolites in the diagnosis of BC. Although the changing trend of tyrosine among different studies were inconsistent, previous research has demonstrated that tyrosine deficiency could result in BC cell growth arrest 69, and inhibition of tumor growth has been confirmed in low phenylalanine and tyrosine diets in an animal study 70. Recent studies also showed that alanine had a significant difference between estrogen receptor positive and estrogen receptor negative breast cancer 71-72. In addition, alanine and valine could result in cell damage by decreasing the activity of manganese superoxide dismutase (MnSOD), thereby converting benign tumor to malignant tumor 73.

Pathway analysis showed that alanine, aspartate and glutamate metabolism played an important role in the development of BC. The down-regulated glutamine indicated that glutamic acid might be accumulated in the body, which promotes the occurrence of BC by enhancing the proliferation of mammary epithelial cells 73 through ATP production and biosynthesis of nucleotides 74. Moreover, the up-regulated glutamic acid via glutaminolysis could maintain the TCA cycle 75. Researchers also observed that the reversibility of glutamine-glutamate was decreasing in MCF-7 cells, implying that BC cells might be partial to the irreversible glutaminase 57. The change of glutamine could be reflected in the fluctuant levels of alanine and aspartic acid through the abnormal transport of ammonia. Higher activities of histidine decarboxylase might result in decreasing histidine since decarboxylation of histidine by this enzyme in the colorectal cancer has been reported 76-77. Therefore, the low concentrations of histidine could be accounted for increased aspartic acid and glutamic acid, which could be converted to oxaloacetic acid and α-ketoglutaric acid, the intermediates of TCA cycle. Aspartic acid has been shown to possess a higher sensitivity for BC compared with gastric and colorectal cancer 32. Therefore, increasing utilization of aspartic acid by BC cells might result in down-regulated aspartic acid and oxaloacetate in the blood. Notably, as a transamination product of aspartic acid, asparagine has a vital influence on the metastasis of BC 78. Furthermore, with the role of modifying the indices of oxidative stress and membrane damage, increased hypotaurine could be potentially linked with BC 79.

### 4.3 Lipid Metabolism

Previous studies had put large efforts to figure out the importance of lipid metabolism in the diagnosis of breast cancer 21, 26, 31, 35, 38-40, 50, 54, 56, but deterministic conclusions are still on the road due to the complexity. It is well known that an increase of choline in the tissue could be a hallmark of aggressiveness breast cancer 80, therefore, excessive consumption of choline could result in its lower blood levels 23, 30, 32, 34. The phenomenon that phospholipid metabolism shares the trend of upregulation integrally 21, 23 could be explained by an enhanced fatty acid (FFA) oxidation to confer limitless growth or survival advantage^81^, mainly through inducing more exalted cell membrane turnover and lipid activity in intracellular signal transduction 82-84. Remarkably, increased phosphocholine has been reported in other cancers, such as lung cancer 85, prostate cancer 86, brain cancer 87, colorectal cancer 88 and cervical cancer 89. Fatty acid synthase (FANS) has also been confirmed to be highly expressed in the MCF-7 cells and tissue of BC patients 90. Linoleic acid, stearic acid and palmitic acid were consistently detected in different studies with elevated levels in BC. Linoleic acid could promote BC via modulating Breast Cancer Susceptibility Gene 1 (BRCA1) 91. Meanwhile, linoleic acid could increase the production of 20-hydroxyeicosatetraenoic acid (12-HETE), 15-hydroxyeicosatetraenoic acid (15-HETE) and prostaglandin E2 92, which helps increase the modulation of adhesion and the membrane fluidity to promote BC 93. Palmitic acid and stearic acid, as two common saturated fatty acids, were confirmed to inhibit insulin metabolism and attenuate insulin signal transduction 94. Furthermore, a previous study demonstrated that a high level of palmitic acid in erythrocyte could increase the risk of BC 95. Acetone, one of ketone bodies, was also suggested to be an aggressive biomarker of BC cells 53. As another important way to provide energy in lipid metabolism, ketone bodies that are promoted by glycolytic stromal cells might provoke tumor growth and metastasis by accelerating oxidative mitochondrial metabolism 96.

## 5. Conclusions

In conclusion, numerous available publications have demonstrated the potential of metabolic profiling applied to the diagnosis of BC. Our review presents that there indeed exist certain metabolisms in BC patients, which could lay foundation for biomarker discovery for BC diagnosis and shed new light into the development and progression of BC. Moreover, with the advantages of convenience and relative non-invasion compared with imaging screening and tissue biopsy, metabolomics may be considered as an applicable tool in the diagnosis of early BC.

## Supplementary Material

Supplementary tables.

## Figures and Tables

**Table 1:** High frequency clinical metabolic biomarkers related to BC diagnosis

No.	Metabolites	Hits	Changing Direction in BC	
			up	down
1	Tyrosine	12	6 serum^15^, ^27^, plasma^17^, tissue^18^, saliva^42^, urine^46^	6 plasma^9^, ^34^, ^41^, serum^14^, ^18^, ^23^
2	Alanine	11	6 saliva^11^, ^42^, serum^15^, plasma^21^, ^41^, urine^43^	5 plasma^9^, ^34^, serum^14^, urine^22^, serum&plasma^30^
3	Glutamic acid	10	6 serum^15^, tissue^16^,^18^, plasma^21^, saliva^17^,^42^	4 plasma^9^, serum^14^, urine^22^, serum&plasma^32^
4	Valine	10	4 saliva^11^, ^42^, serum^15^, plasma^21^	6 plasma^9^, ^17^, ^24^, ^34^, serum^14^, urine^22^
5	Phenylalanine	9	5 serum^15^, tissue^18^, saliva^26^, ^42^, urine^46^	4 plasma^9^, ^34^, ^41^, serum^18^
6	Glutamine	9	4 saliva^11^, serum^15^,tissue^18^, serum&plasma^32^	5 plasma^12^, ^17^, ^24^, ^41^ serum^14^
7	Lysine	9	3 plasma^17^, ^41^, saliva^42^	6 plasma^9^, ^12^, ^24^, serum^14^, ^23^, ^27^
8	Isoleucine	8	4 saliva^11^, serum^15^, plasma^21^, urine^43^	4 serum^14^, plasma^17^, ^34^, serum&plasma^32^
9	Histidine	7	3 serum^15^, tissue^18^, saliva^26^	4 serum^14^, ^23^, ^27^, plasma^41^
10	Choline	7	3 saliva^11^, plasma^12^, tissue^36^	4 serum^23^, serum&plasma^30^, ^32^, plasma^34^
11	Glycine	6	4 serum^15^, tissue^37^, plasma^41^ , saliva^42^	2 urine^22^, serum&plasma^30^
12	Arginine	6	3 serum^10^, saliva^11^, plasma^17^	3 serum^14^, ^15^, plasma^24^
13	Asparagine	6	4 plasma^13^, serum^15^, serum&plasma^32^, urine^43^	2 plasma^9^, serum^27^
14	Proline	6	3 serum^15^, plasma^41^, saliva^42^	3 plasma^13^, serum^14^, ^27^
15	Serine	6	5 saliva^11^, ^42^, serum^15^, plasma^21^, ^41^	1 serum&plasma^30^
16	Creatine	6	3 tissue^18^, ^37^, urine^43^	3 serum^18^, plasma^24^, ^34^
17	Leucine	6	2 saliva^11^, urine^43^	4 plasma^9^, ^34^, serum^14^, urine^22^
18	Tryptophan	6	1 serum^33^	5 serum^14^, ^18^, ^27^, urine^22^, plasma^41^
19	Lactate	6	4 saliva^11^, plasma^17^, ^21^, tissue^37^	2 serum&plasma^32^, plasma^34^
20	Threonine	5	2 plasma^41^, saliva^42^	3 plasma^9^, serum^14^, urine^22^
21	Taurine	5	4 plasma^13^, ^24^, serum^15^, tissue^37^	1 saliva^42^
22	Glucose	5	2 plasma^17^, urine^43^	3 serum^23^, plasma^34^, tissue^37^
23	Aspartic acid	4	3 serum^10^, tissue^16^, saliva^42^	1 serum&plasma^32^
24	Stearic acid	4	4 plasma^13^, ^21^, ^24^, serum^38^	
25	Ornithine	4	2 plasma^41^, saliva^42^	2 plasma^9^, serum^14^
26	Cysteine	4	2 serum^15^, serum&plasma^32^	2 urine^22^, serum^27^
27	Glycerophosphocholine	4	2 plasma^12^, tissue^36^	2 serum&plasma^32^, saliva^42^
28	Pyruvate	3	2 serum&plasma^30^, plasma^34^	1 serum^18^
29	Linoleic acid	3	3 plasma^21^, ^24^, serum^38^	
30	Palmitic acid	3	3 plasma^13^, ^21^, serum^38^	
31	Uracil	3	2 serum&plamsa^32^, urine^43^	1 urine^22^
32	Urea	3	2 urine^43^, ^45^	1 plasma^8^
33	Formate	3	1 urine^43^	2 serum^10^, plasma^17^

**Table 2:** Significant metabolic pathways related to BC diagnosis in clinical researches

Pathway Name	Raw *P*	Holm Adjust	FDR*	Impact
Arginine and proline metabolism	6.69E-09	5.35E-07	5.35E-07	0.70435
Glycine, serine and threonine metabolism	4.27E-08	3.37E-06	1.71E-06	0.53424
Aminoacyl-tRNA biosynthesis	5.59E-07	4.36E-05	9.45E-06	0.22536
Alanine, aspartate and glutamate metabolism	5.61E-07	4.36E-05	9.45E-06	0.85945
Nitrogen metabolism	5.91E-07	4.49E-05	9.45E-06	0.00830
Glutathione metabolism	1.80E-05	0.0013531	2.41E-04	0.34568
beta-Alanine metabolism	2.77E-05	0.0020517	3.17E-04	0.41674
Glycerophospholipid metabolism	6.21E-04	0.0453150	0.006208	0.41257
Cysteine and methionine metabolism	0.001042	0.0750390	0.009264	0.50502
Glyoxylate and dicarboxylate metabolism	0.001342	0.0952670	0.010734	0.46883
Citrate cycle (TCA cycle)	0.003391	0.2373800	0.022608	0.42880
Taurine and hypotaurine metabolism	0.003391	0.2373800	0.022608	0.46583
Pantothenate and CoA biosynthesis	0.003890	0.2645400	0.023940	0.25300

*FDR: false discovery rate

**Table 3:** The potential for metabolite markers in the diagnosis of BC

Ref.	Sample	Sensitivity	Specificity	AUC	Potential Biomarker(s)	Note
Baowen Yuan 9(2019)	plasma	-	-	0.870	all significant metabolites	BC vs HC(training)
		-	-	0.800	all significant metabolites	BC vs HC(validation)
Paniz Jasbi 13(2019)	plasma	80.00%	75.00%	0.890	all significant metabolites	BC vs HC
		-	-	0.760	proline	BC vs HC
		86.00%	75.00%	0.870	all significant metabolites	EBC vs HC
Dan Tudor Eniu 14(2019)	serum	83.33%	76.92%	0.850	isoleucine	BC vs HC
		66.67%	92.31%	0.850	tryptophan	BC vs HC
Xinyang Wang 15(2018)	serum	-	-	0.924	glutamic acid	BC vs HC
		-	-	0.901	taurine	BC vs HC
		-	-	0.749	ethylmalonic acid	BC vs HC
Shankar Suman 17(2018)	plasma	-	-	0.818	β-glucose	BC vs HC
		-	-	0.780	α-glucose	BC vs HC
		-	-	0.780	lactate	BC vs HC
		-	-	0.697	hydroxybutyrate	BC vs HC
		-	-	0.652	N-acetyl glycoprotein	BC vs HC
		-	-	0.627	lysine	BC vs HC
Tushar H. More 18(2018)	tissue	-	-	0.970	guanine	IDC vs HC
		-	-	0.830	tyrosine	IDC vs BE
		-	-	0.960	tyrosine	IDC vs HC
	serum	-	-	0.980	ascorbic acid	IDC vs HC
		-	-	0.830	uridine diphosphate	IDC vs BE
Mónica Cala 22(2018)	urine	93.50%	86.20%	0.915	dimethylheptanoylcarnitine +succinic acid	BC vs HC
Mariona Jové 24(2017)	plasma	100.00%	100.00%	1.000	C26H43ClN4S3	BC vs HC
		100.00%	100.00%	1.000	C26H51N5O4	BC vs HC
		100.00%	100.00%	1.000	C9H16O3S	BC vs HC
		100.00%	100.00%	0.999	C23H30N2S	BC vs HC
		100.00%	100.00%	0.995	caproic acid	BC vs HC
		100.00%	90.00%	0.952	taurine	BC vs HC
		90.00%	90.00%	0.959	stearamide	BC vs HC
		100.00%	90.00%	0.935	linoleic acid	BC vs HC
Naila Irum Hadi 25(2017)	serum	96.00%	100.00%	0.990	all significant metabolites	BC vs HC
Liping Zhong 26(2016)	saliva	92.60%	91.70%	0.929	MG(0:0/14:0/0:0)	BC vs HC
		77.80%	100.00%	0.920	LysoPC (18:1)	BC vs HC
		81.50%	91.70%	0.920	LysoPC (22:6)	BC vs HC
Qingjun Wang 27(2016)	serum	90.30%	87.40%	0.944	all significant metabolites	BC vs BE(HC)
Takahiro Takayama 28(2016)	saliva	68.90%	74.40%	0.744	spermine.	BC vs HC
Luisa Matos Do Canto 29(2016)	ductal fluid	90.70%	88.40%	0.956	all significant metabolites	BC vs HC
Sijia Huang 30(2016)	plasma	-	-	0.986	all significant metabolites	BC vs HC(training)
		-	-	0.995	all significant metabolites	EBC vs HC(training)
		-	-	0.923	all significant metabolites	BC vs HC(testing)
		-	-	0.905	all significant metabolites	EBC vs HC(testing)
	serum	-	-	0.995	all significant metabolites	BC vs HC(validation)
		-	-	0.902	all significant metabolites	EBC vs HC(validation)
Guoxiang Xie 32(2015)	plasma	100.00%	100.00%	1.000	aspartic acid	BC vs HC(training)
		100.00%	94.30%	0.996	glycerolphosphate	BC vs HC(training)
	plasma	85.40%	95.10%	0.935	aspartic acid	BC vs HC(validation)
		95.10%	93.20%	0.971	glycerolphosphate	BC vs HC(validation)
Yunping Qiu 35(2013)	plasma	98.10%	96.00%	-	LysoPC a C16:0, PC ae C42:5 and PC aa C34:2	BC vs HC
Tone F. Bathen 37(2013)	tissue	91.00%	93.00%	-	all significant metabolites	BC patientstumor tissue vs non-involved adjacent tissue
Wuwen Lv 38(2012)	serum	82.80%	85.30%	0.892	C16:0	BC vs HC
		89.70%	85.00%	0.925	C16:0	BC vs BE
Yohei Miyagi 41(2011)	plasma	-	-	0.778	all significant metabolites	BC vs HC
		-	-	0.813	all significant metabolites	Stage 0 BC vs HC
		-	-	0.754	all significant metabolites	Stage I BC vs HC
		-	-	0.786	all significant metabolites	Stage II BC vs HC
		-	-	0.755	all significant metabolites	Stage III BC vs HC
Masahiro Sugimoto 42(2010)	saliva	-	-	0.973	all significant metabolites	BC vs HC
Carolyn M. Slupsky 43(2010)	urine	100.00%	93.00%	-	all significant metabolites	BC vs HC
Hojung Nam 45(2009)	urine	-	-	0.790	all significant metabolites	BC vs HC

**Table 4:** High frequency cellular metabolic biomarkers related to BC diagnosis

No.	Metabolites	Hits	Changing direction in BC	
			up	down
1	Leucine	2		2^47^, ^51^
2	Isoleucine	2		2^47^, ^51^
3	Valine	2		2^47^, ^51^
4	Phenylalanine	2		2^47^, ^51^
5	Glutamine	2		2^47^, ^57^
6	Glucose	2		2^53^, ^57^
7	Phosphocholine	2	2^53^, ^56^	
8	3-Methylcytidine	2	2^49^, ^52^	
9	5-Methyluridine	2	2^49^, ^52^	

**Table 5:** Significant metabolic pathways related to BC diagnosis in cellular researches

Pathway Name	Raw *P*	Holm Adjust	FDR^*^	Impact
Aminoacyl-tRNA biosynthesis	6.23E-08	0.00000	4.98E-06	0.11268
Nitrogen metabolism	9.91E-07	0.00008	3.97E-05	0.00830
Glycerophospholipid metabolism	1.66E-05	0.00129	4.42E-04	0.32074
Glycolysis or Gluconeogenesis	7.21E-05	0.00555	0.0014422	0.14226
Alanine, aspartate and glutamate metabolism	3.62E-04	0.02753	0.0057956	0.51757
Valine, leucine and isoleucine biosynthesis	5.79E-04	0.04345	0.006910	0.06148
Glycine, serine and threonine metabolism	6.05E-04	0.04474	0.006910	0.13604
Cyanoamino acid metabolism	0.0015061	0.10995	0.015061	0.00000

*FDR: false discovery rate
