# Supplementary material for: Application of metabolomics in the diagnosis of breast cancer: a systematic review
Source: J Cancer. 2020 Feb 10;11(9):2540–51. doi: 10.7150/jca.37604 (PMC7066003; doi:10.7150/jca.37604)
Supplement: Supplementary file 1 — Supplementary tables. [file jcav11p2540s1.pdf]

Supplementary Table S1

Ref.	First Author (Year)	Case (Sample Size)	Control (Sample Size)	Sample	Detecting Instrument	Targeted/Untargted	Up-regulated Metabolites	Down-regulated Metabolites
8	Hua Zhao (2019)	BC patients(358)	healthy control(138)	plasma	UHPLC-MS<br>GC-MS	Untargeted	thioproline, inosine, 3-hydroxyoctanoate, 3-hydroxybutyrate, stearate (18:0), linoleate (18:2n6), dihomo-linoleate, 10-nonadecenoate(19:1n9)	cysteine S-sulfate, threonylphenylalanine, sphingomyelin, bilirubin, glucuronate, 1-(1-enyl-stearoyl)-2-dihomo-linolenoyl-GPE, urea, 3-methylxanthine, 1-palmitoyl-GPA (16:0)
9	Baowen Yuan (2019)	BC patients(189)	healthy control(150)	plasma	UHPLC-MS/MS<br>FIA-MS/MS	Targeted	acetylcarnitine C2, C14:1	alanine, asparagine, glutamate, histine, leucine, lysine, methionine, ornithine, phenylalanine, threonine, trptophan, tyrosine, valine, kynurenine, methionine sulfoxide, acetylcarnitine C3
10	Matthias Pietzke (2019)	BC patients(68)	healthy control(50)	serum	GC-MS<br>LC-MS	Targeted	sernine, aspartate, arginine	formate, sarcosine
11	Takeshi Murata (2019)	IDC patients(101)	healthy control(42)<br>(BE patients 2)	saliva	LC-MS<br>CE-MS	Untargeted	spermine, N1-acetylspermine, leucine, glutamine, serine, spermidine, isoleucine, N1-acetylspermidine, cadaverine. butanoate, carnitine, succinate, theonine, arginine, lactate, valine, 1,3-diaminopropane, Ru5P, gamma-butyrobetaine, N-acetylneuraminate, alanine, propionate, choline, DHAP, 5-aminovalerate, GABA	
12	Xiao Luo (2018)	BC patients(60)	healthy control(40)	plasma	LC-MS	Untargeted	glycerophosphocholine, phenyllactic acid, pentosidine, epinephrine glucuronide, homoanserine, nicotinuric acid, choline	glutamine, lysine, glycolaldehyde, sphingosine 1-phosphate, phenylglyoxylic acid, butyrylcarnitine, 3-pyridylacetic acid, 3-indolepropionic acid, stearoylcarnitine, linoleyl carnitineic acid, bilirubin, indoleacrylic acid
13	Paniz Jasbi (2019)	BC patients(102)	healthy control(99)	plasma	LC-MS/MS	Targeted	hypoxanthine, palmitic acid, stearic acid, acetylglycine, nonadecanoic acid, asparagine, citraconic acid, 2-aminoisobutyric acid, 3-indolepropionic acid, N,N'-dicyclohexylurea, taurine, decanoylcarnitine, urocanic acid	proline, myoinositol, 2-hydroxybenzoic acid, gentisic acid, 2,3-dihydroxybenzoic acid, 5-aminolevulinic acid, pantothenic acid, cytidine, 4-pyridoxic acid, TMAO, agmatine, indole-3-acetic acid, indole, betaine, glycocyamine, xanthine, 3-indoxylsulfate
14	Dan Tudor Eniu (2019)	BC patients(30)	healthy control(26)	serum	UHPLC-QTOF-(ESI+)-MS	Targeted		arginine, alanine, isoleucine, tyrosine, tryptophan, histidine, leucine, lysine, threonine, valine, glutamine, glutamic acid, proline, amino adipic acid, ornithine, citrulline
15	Xinyang Wang (2018)	BC patients(34)	healthy control(44)	serum	UPLC/MS-MS<br>GC-MS	Targeted	alanine, aminobutyric acid, asparagine, creatinine, cysteine, dimethylglycine, glycine, glutamic acid, glutamine, histidine, isoleucine, methionine, proline, valine, phenylalanine, serine, taurine, tyrosine	γ-aminobutyric acid, arginine

16	Nobuhiro Torata (2018)	BC patients(84)	healthy control(35)	tissue	MALDI MSI	Targeted	adenosine diphosphate, adenosine monophosphate, adenosine triphosphate, aspartate, citrate, deoxycytidine diphosphate, fructose 1,6-bisphosphate, glutamate, glutathione, glutathione disulfide, guanosine diphosphate, N-acetylaspartate, NADH, UDP-glucose, DP-N-acetylglucosamine, UDP, UMP	
17	Shankar Suman (2018)	BC patients(72)	healthy control(50)	plasma	1H NMR	Untargeted	tyrosine, lipid, glucose, lactate, hydroxybutyrate, glutamate, lysine, arginine, lipoprotein	formate, glutamine, NAG, valine, isoleucine
18	Tushar H. More (2018)	IDC(76), benign(33) and control(33)		serum	LC-/MRM-MS GC-MS	(LC-/MRM-MS) Targeted (GC-MS) Untargeted	α-ketoglutaric acid	ascorbic acid, tryptophan, tyrosine, phenylalanine, uric acid, UDP, creatine, pyruvate
		IDC(24), benign(24) and normal(24)		tissue			histidine, glutamine, tyrosine, creatine, phenylalanine, lactic acid, adonitol, glutamic acid	3,7-cholest-5-ene
19	Mengzhe Guo (2018)	BC patients(10)	healthy control(10)	urine	HPLC-ESI-MS/MS	Targeted	5-(formyl)-2'-deoxycytidine, 5-(carboxyl)-2'-deoxycytidine	5-(hydroxymethyl)-2'-deoxycytidine
20	Carina Cavaco (2018)	BC patients(66)	healthy control(40)	saliva	HS-SPME/GC-MS	Untargeted	acetic acid, propanoic acid	3-methyl-pentanoic acid, 4-methyl-pentanoic acid, phenol, 1,2-decanediol
21	Mo´nica P. Cala (2018)	BC patients(58)	healthy control(29)	plasma	GC-MS NMR LC-MS	Untargeted	valine, alanine, isoleucine, serine, glutamic acid, 4-hydroxyproline, 2-hydroxybutyric acid, 3-hydroxybutyric acid, glycerol, myristic acid, palmitoleic acid, palmitic acid, linoleic acid, oleic acid, arachidonic acid, decanoylcarnitine, decenoylcarnitine, dodecenoylcarnitine, laurylcarnitine, linoleyl carnitine, myristoylcarnitine, oleoylcarnitine, palmitoylcarnitine, tetradecadiencarnitine, tetradecenoylcarnitine, 11’-carboxy-γ-tocotrienol, 9’-carboxy-γ-tocotrienol, γ-homolinolenic acid, adrenic acid, arachidonic acid, docosapentaenoic acid, 12-HETE, stearic acid, lactate, lipids	pyruvic acid, cPA(18:0)

22	Mónica Cala (2018)	BC patients(31)	healthy control(29)	urine	GC-MS<br>LC-QTOF/MS	Untargeted	PG(O-36:1), PA(O-16:0), PC(34:1), DG(32:0),<br>PA(37:6), PA(33:3)	valine, alanine, leucine, threonine, glycine, β-alanine, glutamic acid, cysteine, tryptophan, glycolic acid, malonic acid, maleic acid, succinic acid, uracil, aminomalonic acid, N-methyl-glutamic acid, N-acetylglutamic acid, homovanillic acid, 4-hydroxymandelic acid, hippuric acid, citric acid, adenine, gulonic acid-lactone, indoleacetic acid, 5-methylthioadenosine, cetohexoses, sucrose, turanose, dimethylheptanoylcarnitine, decenoylcarnitine, decatrienoylcarnitine, decadienoylcarnitine, octenoylcarnitine, LPE(18:2), LPE(20:4), LPC(14:1), glycerol 3-phosphate
23	Jianping Zhou (2017)	BC patients(11)	healthy control(11)	serum	NMR	Untargeted	lipid	choline, glucose, histidine, vailne, lysine, acetate, tyrosine, glutamic
				urine			citrate	phenylacetylglycine, guanidoacetate
24	Mariona Jové (2017）	BC patients(91)	healthy control(20)	plasma	LC-MS/MS	Untargeted	2-hydroxy-3-methylbutyric acid, 2-hydroxy-3-methylpentanoic acid, 3-methylglutaric acid, 7-ketocholesterol, chenodeoxycholic acid, cytidine, DL-pipecolic acid, dopamine, homocystine, inosine diphosphate, linoleic acid, myristic acid, oleamide, stearic acid, taurine, threonate, uric acid	2-methylhippuric acid, 2-octenoic acid, 3-hydroxyanthranilic acid , 4-acetamidobutanoate, 5-b-cholestane-3a, 7a, 12a-triol, 5α-androstane-3,17-dione, 7α-hydroxy-cholesterol, caproic acidcortisol, cortisone, creatine, glutamine, hippuric acid, L-arginine, L-lysine, L-valine, N-oleoyl-D-erythroSphingosine(C18:1 ceramide), retinoic acid
25	Naila Irum Hadi (2017)	pre-operative BC patients(152)<br>neoadjuvant BC	healthy control(155)	serum	GC-MS	Untargeted	tetradecane, α-D-glucopyranoside, methyl stearate, 1-4-benzene	dimethydodecane, D-galactose, α-glyceryl stearate
26	Liping Zhong (2016)	BC patients(30)	healthy control(25)	saliva	HILIC<br>RPLC UPLC-ESI-MS	Untargeted	lysoPC(18:1), PS(14:1/16:1), lysoPC(16:0), lysoPC(22:6), MG(0:0/14:0/0:0), lysoPE(18:2/0:0), PC(18:0/16:0), phenylalanine, citrulline, histidine, N-acetylneuraminic acid, PE(22:0/20:4), 4-hydroxyphenylpyruvic acid	lysoPC(18:2), palmitic amide, phytosphingosine acetylphenylalanine, propionylcholine
27	Qingjun Wang (2016)	early BC patients(258)	BE control(81)<br>healthy people(78)	serum	MS	Targeted	propionylcarnitine, acetylcarnitine, tyrosine	cysteine, proline, asparagine, piperamide, homocysteine, tryptophane, lysine, histidine
28	Takahiro Takayama (2016)	BC patients before(111) and after operation(80)	healthy control(61)	saliva	UPLC-MS/MS	Targeted	SPM(spermine), CAD(cadaverine), Ac-SPM(N1-acetyl-spermine), N1-Ac-SPD(N1-acetyl-spermidine), N8-Ac-SPD(N8-acetyl-spermidine), DAc-SPD(N1N8-diacetyl-spermidine),DAc-SPM(N1N12-diacetyl-spermine), Ac-PUT（N1-acetyl-putrescine）	

29	Luisa Matos Do Canto (2016)	BC patients(43) affected breast vs unaffected control		ductal fluid	UPLC-QTOF/MS	Untargeted	angustifoline, phosphatidyl glycerol (12:0/0:0)	N-acetyl-dtryptophan, 1,2,3,4-tetrahydroisoquinoline, gluconolactone, phosphatidyl glycerol (18:0/0:0), 9-amino-nonanoic acid, hydrocortisone butyrate, ceramide (d18:2/22:0), pentaethylene glyco, peroxyacetic acid uroporphyrin III
30	Sijia Huang (2016)	BC patients(106)	healthy control(61)	serum plasma	LC-TOFMS GC-TOFMS	Untargeted	hypotaurine, pyruvate	succinate, choline, serine, glycine, alanine
31	Min Cui (2016)	IDC paients(20)	healthy control(20)	serum	LC/MS	Untargeted	LPE(18:1/0:0), LPE(18:0/0:0), LPE(22:0/0:0), sphingomyelin(d18:0/20:0), PC(38:0), glutaconylcarnitine, tryptophyl-leucine, tyrosyl-aspartate, aspartyl-phenylalanine, diglyceride(20:1/14:1/0:0), diglyceride(22:5/22:0/0:0)	ceramide(d18:1/26:0), ceramide(d18:1/20:0), ceramide(d18:1/23:0), LPC(18:0/0:0)
32	Guoxiang Xie (2015)	BC patients(35)	healthy control(35)	serum plasma	LC-TOFMS GC-TOFMS	Untargeted	cystine, glutamine, cysteine, hypotaurine, asparagine, butyryl carnitine, propionyl carnitine, glyoxylate, uracil	aspartate, glutamate, 5-oxoproline, isoleucine, N-acetyl glutamine, aminoacetone, delta-hydroxylysine, glycerolphosphate, glycerophosphocholine, arachidonate, octadecanoate, nicotinurate, choline, α-ketoglutarate, oxaloacetate, 4-hydroxy-2-oxoglutarate, lactate, malate, pentosidine, quinate, epinephrine, glucuronide
33	ZhuGen Cao (2015)	BC patients(20)	healthy control(50)	serum	ESI-QTOF-MS	Targeted	tryptophan	
34	Batur Mamtimin (2014)	BC patients(35)	healthy control(35)	plasma	NMR	Untargeted	VLDLs, unsaturated lipids, pyruvate, carnitine	creatine, creatinine, lactate, isoleucine, leucine, valine, alanine, glycoprotein, 1-methylhistidine, tyrosine, phenylalanine, myo-inositol, scyllo-inositol, choline, α-glucose, β-glucose
35	Yunping Qiu (2013)	BC patients(55)	healthy control(25)	plasma	ESI-MS/MS	Targeted	PC ae C36:2, PC aa C38:6, PC aa C40:6, PC aa C34:2, PC aa C38:0, PC ae C34:2, SM (OH) C22:2, SM (OH) C14:1, SM (OH) C16:1, SM (OH) C22:1, SM C20:2, C4	PC ae C40:3, PC ae C38:3, PC ae C40:4, PC ae C38:1, PC ae C42:4, PC ae C40:5, PC ae C40:2, PC ae C44:3, PC ae C38:2, PC ae C42:1, PC aa C40:3, PC aa C40:2, PC aa C40:4, PC aa C42:5, lysoPC a C16:0, lysoPC a C18:0, lysoPC a C20:4, lysoPC a C18:1, lysoPC a C17:0, lysoPC a C20:3, lysoPC a C28:0, lysoPC a C16:1, lysoPC a C24:0, lysoPC a C26:0, PC aa C42:4，PC ae C42:5, PC ae C40:3，PC ae C40:2
36	Maria Chiara Mimmi (2013)	BC patients(3)	healthy control(3)	tissue	LC-ESI-MS/NMR	Targeted	choline, phosphocholine, glycerophosphocholine	
37	Tone F. Bathen (2013)	BC patients(228) tumor tissue vs non-involved adjacent tissue		tissue	HR MAS MRS	Untargeted	phosphocholine, glycine, taurine, creatine, lactate, ascorbate	glucose

38	Wuwen Lv<br>(2012)	BC patients(40)	BE patients(40)<br>healthy control(34)	serum	GC-MS	Targeted	(BC vs BE/HC)<br>palmitic acid, stearic acid, linoleic acid, n-6, total free fatty acid	(BC/BE vs HC)<br>C12:0<br>(BC vs HC)<br>C18:3, C20:5, C22:5
39	Jiang Huang<br>(2012)	postmenopausal BC(86)	healthy control(36)	urine	UFLC-MS/MS	Targeted	estrone, 17β-estradiol, 2-hydroxyestradiol, 4-hydroxyestradiol	16α-hydroxyestrone, 2-methoxyestradiol
40	Scarlet F. Brockm€oller<br>(2012)	BC patients(228)	healthy control(228)	tissue	GC-MS, LC-MS	Targeted	sn-glycerol-3-phosphate, PC(34:3), PC(36:4), PC(34:4e), TG(16:0/16:1/18:1), PC(38:5e), PC(36:5), Cer(d18:1/17:0), PC(36:2), PE(36:2), PE(P-18:0:22:5), PC(38:6), PC(34:2), PE(P-38:5), PC(32:1), PC(38:5), TG(49:3), SM(d18:1/14:0), PC(18:0/22:6), PI(18:0/20:4), PC(P-18:0/20:4)/PC(O18:2/20:4)	
41	Yohei Miyagi<br>(2011)	BC patients(196)	healthy control(976)	plasma	HPLC-ESI-MS	Targeted	glycine, alanine, ornithine, threonine, serine, proline, lysine	glutamine, tyrosine, tryptophan, phenylalanine, histidine
42	Masahiro Sugimoto<br>(2010)	BC patients(30)	healthy control(87)	saliva	CE-TOF-MS	Untargeted	cadaverine, glycine, alanine, putrescine, phenylalanine, tyrosine, ethanolamine, serine, α-aminobutyric acid, leucine+isoleucine, proline, lysine, ornithine, aspartic acid, valine, glutamic acid, threonine	glycerophosphocholine, taurine, piperideine
43	Carolyn M. Slupsky<br>(2010)	BC patients(48)	healthy control(72)	urine	NMR	Targeted	creatine, acetate, succinate, latate, pyroglutamate, formate, isoleucine, sucrose, leucine, asparagine, urea, glucose, ethanolamine, dimethylamine, 4-hydroxyphenylacetate, creatinine, alanine, hippurate, uracil, vailne, trans-aconitate	
44	Han Min Woo<br>(2009）	BC patients(10)	healthy control(22)	urine	GC-MS<br>LC-MS	Untargeted	8-hydroxy-2-deoxyguanosine, 5-hydroxymethyl-2-deoxyuridine, 11-ketoandrosterone	androsterone, 16α-hydroxydehydroepiandrosterone
45	Hojung Nam<br>(2009)	BC patients(50)	healthy control(50)	urine	GC-MS	Untargeted	urea	homovanillate, 4-hydroxyphenylacetate, 5-hydroxyphenylacetate
46	Yanhua Chen<br>(2009)	BC patient(20)<br>(16 had IDC)	healthy control(18)	urine	RRLC-MS/MS	Untargeted	tyrosine, phenylalanine, trytophan, nicotinuric acid, kynurenic acid, indolelactic acid, dimethylarginine, pantothenic acid, succinyladenosine, dimethylguanosine, apronal, threonylcarbamoyl adenosine	

Supplementary Table S2

Ref.	First Author (Year)	BC Cell Lines	Normal Control	Sample	Detecting Instrument	Targeted/Untargted	Up-regulated Metabolites	Down-regulated Metabolites
47	Siqi Du (2019)	MCF-7	MCF-10A	cell	HPLC-MS/MS	Untargeted	aspartic acid, serine, asparagine	leucine, isoleucine, valine, phenylalanine, glutamine
48	Catarina L. Silva (2017)	T-47D, MDA-MB-231, MCF-7	HMEC	cell	HS-SPME GC-MS	Untargeted	2-pentanone, 2-heptanone, 3-methyl-3-buten-1-ol, ethyl acetate, ethyl propanoate, 2-methyl butanoate	
49	Lucas Willmann (2016)	BT-474, MDA-MB-231 MDA-MB-453	MCF-10A	cell	LC-MS	Untargeted	1-methylinosine, 3-methylcytidine, 5-methyluridine, N2-methylguanosine, N6-methyl-N6-threonylcarbamoyladenosine, N6-threonylcarbamoyladenosine	prostaglandin F1α, hypoxanthine
50	Hye-Youn Kim (2016)	MCF-7, MDA-MB-231	MCF-10A	cell	GC-MS DI-MS	Untargeted	guanine, PS(18:1/22:4), PE(18:1/20:4), PI(16:0/18:1), PS(17:0/18:1), PC(16:1/18:1), PC(18:1/18:2), PC(18:1/18:1), PC(18:2/20:3), PC(18:0/20:4), PC(18:1/20:0), PC(18:1/22:5), PE(18:1/20:4), PE(18:1/22:4), PS(16:1/18:1), PS(16:0/18:1), PS(16:0/18:0), PS(17:0/18:1), PS(18:0/18:0), PS(18:1/20:0), PS(18:0/22:6), PS(18:1/22:0), PI(16:0/16:1), PI(16:1/18:1), PI(18:1/20:4)	xanthine, glucose-6-phosphate, mannose-6-phosphate
32	Guoxiang Xie (2015)	MCF-7	MCF-10A	cell	LC-TOFMS GC-TOFMS	Targeted	aspartate	
51	Lucas Willmann (2015)	MDA-MB-231, MDA-MB-453, BT-474	MCF-10A	cell	GC-EI-MS	Untargeted	gluconic caid, 3-phosphoglyceric acid, pyruvic acid, taurine	isoleucine, tyrosine, valine, tryptophan, phenylalanine, leucine
52	Lucas Willmann (2015)	BT-474, MDA-MB-231, MD-MBA-453	MCF-10A	cell	LC-MS	Targeted	pseudouridine, 3-methylcytidine, 1-methyladenosine, 5-methyluridine, 1-methylguanosine, xanthosine, N4-acetylcytidine, N6-threonyl-carbamoyladenosine, 2-methylthio-N6-threonylcarbamoyladenosine, N6-Methyl-N6-threonylcarbamoyladenosin	
53	Roberta M. Maria (2015)	MCF-7, MDA-MB-231	MCF-10A	cell	1H HR-MAS NMR	Untargeted	phosphocholine, acetone	glucose, ethanol
54	Dória, M. Luísa （2013)	T47-D, MDA-MB-231	MCF-10A	cell	TLC-MS	Untargeted	alkylacyl PCs, phosphatidylinositol, PI (18:0/18:1), PI (18:1/18:1 and 18:2/18:0), PC (14:0/18:1 and 16:0/16:1), PI (16:0/18:1 and 16:1/18:0)	PI (20:4/18:0)
55	M. Luı ´sa Do´ria (2012)	MC4-L5, MC4-L2	EpH4	cell	TLC-MS ESI–MS	Targeted	sphingomyelin, phosphatidylinositol	phosphatidylethanolamine
56	Maria D. Cao (2012)	MCF-7, MDA-MB-231	MCF-12A	cell	HR MAS MRS	Untargeted	phosphocholine, total choline	glycerophosphocholine
57	Adam L. Meadows (2008)	MCF-7	48R HMECs	cell	GC-MS	Untargeted	lactate	glucose, glutamine
